# Incidence and Predictors of Symptom-Arrhythmia Association During Outpatient Holter Monitoring

**DOI:** 10.7759/cureus.96201

**Published:** 2025-11-06

**Authors:** Avinash Saraiya, Douglas R Corsi, Saaniya Farhan, Cameo Hazlewood, Grace Qiu, Behzad B Pavri

**Affiliations:** 1 Department of Internal Medicine, University of Maryland Medical Center, Baltimore, USA; 2 Internal Medicine, Rutgers Robert Wood Johnson Medical School, New Brunswick, USA; 3 Internal Medicine, Sidney Kimmel Medical College at Thomas Jefferson University, Philadelphia, USA; 4 Cardiology, Thomas Jefferson University Hospital, Philadelphia, USA

**Keywords:** arrhythmia, cardiac monitoring, electrophysiology, holter monitoring, palpitation

## Abstract

Introduction: Reported symptomatic activations by patients who are prescribed Holter monitors (Mortara Instrument, Baxter, Illinois, USA) often do not correlate with true arrhythmia, and recorded ectopy is often asymptomatic. The goal of this study is to describe the prevalence of symptom-arrhythmia association and its relation with age, sex, and underlying co-morbidities.

Methods: In this retrospective chart review, extended Holter recordings (analyzable data >72 hours) from 100 consecutive patients (April to July, 2023) were selected. Patients activated the monitor when they experienced an arrhythmia-related symptom. Accuracy was defined as the percent of activations associated with arrhythmia (atrial premature beat, ventricular premature beat, supraventricular tachycardia, ventricular tachycardia, 2nd/3rd degree atrioventricular block, or sinus exit block). We defined “symptom-arrhythmia association” as accuracy greater than zero.

Results: For 100 patients (mean age: 55±18, 27% male), the average accuracy was 22%. Of the 100 patients, 42 had a symptom-arrhythmia association. On univariate analyses, patients with symptom-arrhythmia association were more likely to be older, have a lower left ventricular ejection fraction (LVEF), and be on cardioactive medications. None of these correlations was significant after multivariable adjustment.

Conclusions: Symptom-arrhythmia association is poor in humans. There are no significant differences by sex, percent Caucasians, presence of mitral valve prolapse, or the number of symptomatic activations in those with symptom-arrhythmia association versus those without.

## Introduction

Ambulatory monitoring devices are frequently prescribed to detect possible associations between patient symptoms and the presence or absence of an arrhythmia in patients experiencing intermittent or fleeting symptoms such as palpitations, dizziness, or syncope. Yet, it is commonly observed that reported symptomatic activations by patients who are prescribed Holter monitors often do not correlate with true arrhythmias, and recorded ectopy is often asymptomatic [1-3]. Common perceptions exist that certain patient groups (e.g., female patients or patients with frequent activations) have poorer symptom-arrhythmia associations, whereas patients with certain comorbidities (such as mitral valve prolapse) may be more “internally aware,” but these associations have not been rigorously studied [4]. Studies have reported the presence of structural heart disease, lower ejection fraction, and advanced age as significant predictors of higher diagnostic yield when using Holter recordings for the evaluation of potentially arrhythmogenic symptoms [5,6]. However, the true prevalence of symptom-arrhythmia correlation and associations with age, sex, and underlying co-morbidities is not well described.

Timely and accurate diagnosis of the arrhythmogenic etiology of various symptoms carries significant clinical implications, as the underlying pathologies necessitate different therapeutic plans. At the same time, the absence of arrhythmia during symptomatic episodes can reassure patients of a non-arrhythmogenic etiology and avoid unnecessary additional investigation. The efficacy of today’s extended ambulatory monitoring devices equipped with patient-activated symptom recorders is well established, but there remains room for improvement [7]. It is therefore beneficial to identify predictors of symptom-arrhythmia association that may help inform Holter monitor prescribing practices.

We sought to identify the overall likelihood of symptoms being associated with true arrhythmia. We secondarily attempted to uncover clinically useful age cutoffs at which patients have low or high associations of symptoms and arrhythmia. Finally, we sought to identify patient characteristics associated with the presence or absence of symptom-arrhythmia association.

## Materials and methods

In this retrospective observational study, Holter recordings from 100 consecutive patients who underwent Holter recordings between April 2023 and July 2023 were selected for analysis. Subjects were selected if there were at least two or more symptomatic activations with analyzable Holter recordings. Activations associated with excessive recording artifacts that rendered the electrocardiogram (ECG) unanalyzable were excluded. Patients with known paroxysmal or persistent atrial fibrillation or flutter were excluded from the study. Repeat Holters on the same patient were excluded. All Holter recordings were obtained using Philips Extended Holter ePatch and analyzed on Philips BioTel Heart scanner (Malvern, Pennsylvania). Patients were instructed to activate the monitor when they experienced arrhythmia-related symptoms (palpitations, chest pain, shortness of breath, dizziness, etc.). The Holter report included the associated rhythm strip, which was then individually interpreted. The report also included the computer-generated total of atrial and ventricular ectopy as the percentage of total QRS complexes. Accuracy was defined as the percentage of symptomatic activations that were associated with a true arrhythmia for each patient. A “true arrhythmia” was any rhythm other than sinus rhythm with or without respirophasic sinus arrhythmia and included the following: atrial premature beats, ventricular premature beats, non-sustained supraventricular tachycardia, non-sustained ventricular tachycardia, second or third degree AV block (that was not prevalent throughout the recording), or sinus exit block/sinus pause. We defined “symptom-arrhythmia association” as accuracy greater than zero.

The 10-second ECG sample surrounding the patient activation was evaluated for the presence of arrhythmia. In addition, we also visually scanned the 40 seconds of slower-speed ECG strip “surrounding” the point of symptomatic activation in order to accommodate the possibility of delayed recognition and activation of symptom markers in older patients.

Statistical analysis

Patient characteristics were reported as mean (standard deviation) or percentage as appropriate. We used Shapiro-Wilk tests to assess the normality of the data. We conducted chi-square tests to compare categorical factors by age, symptom-arrhythmia association, and sex. As deviance from normality was present, we conducted Mann-Whitney U (or Wilcoxon rank sum) tests to compare continuous variables by age, symptom-arrhythmia association, and sex. Logistic regression models were used to assess the relationship between predictor variables (age, left ventricular ejection fraction, use of cardioactive medications) and the presence of symptom-arrhythmia association. Models were sequentially adjusted for sex, race, duration of Holter analysis, comorbidities (mitral valve prolapse and coronary artery disease), and ectopy percent (computer-generated total of atrial and ventricular ectopy percent). We calculated odds ratios, 95% confidence intervals (CI), and p-values for all models. We plotted receiver operating characteristic (ROC) curves and calculated the area under the curve (AUC) for our model using age as a classifier to the presence of symptom-arrhythmia association. Optimal cutoff was determined using the Youden Index. For all models, the threshold for significance was set at alpha < 0.05. All analyses were run in RStudio R 4.2.1.

## Results

For the 100 patients (mean age: 55 ± 18, range: 21-99), the mean recording time was 194 hours with a mean of 5.7 analyzable activations per patient. Baseline characteristics are detailed in Table 1 in the left column. The average accuracy by patient was 22%; i.e., the average likelihood of a true arrhythmia being present during a symptomatic activation for a particular patient was 22%. Older patients had a higher average accuracy compared to younger patients (50% accuracy in patients over 70 vs. 4% in patients under 35). There was no association between age and the number of symptomatic activations (R^2^=0.0016).

**Table 1:** Patient characteristics by the presence of symptom-arrhythmia association The test statistic indicates the Wilcoxon Rank Sum test (W statistic) or the Chi-square test statistic when comparing between continuous or categorical variables, respectively.

	Entire Group	Patients WITHOUT Symptom-Arrhythmia Association	Patients WITH Symptom-Arrhythmia Association	p-value	Test statistic
N	100	58	42	-	-
Age (years)	55 ± 18	50 ± 18	62 ± 15	<0.001	686
Sex (Female), n (%)	73 (73)	46 (79)	27 (64)	0.149	2.08
Race (Caucasian), n (%)	66 (66)	36 (62)	30 (71)	0.447	0.580
Coronary Artery Disease, n (%)	17 (17)	7 (12)	10 (24)	0.203	1.62
Hypertension, n (%)	39 (39)	23 (40)	16 (38)	1.00	0.00
Diabetes Mellitus, n (%)	11 (11)	5 (9)	6 (14)	0.569	0.325
Chronic Kidney Disease, n (%)	12 (12)	8 (14)	4 (10)	0.736	0.113
Left Ventricular Ejection Fraction (%)	60 ± 11	63 ± 7	56 ± 14	0.005	1184
Presence of Mitral Valve Prolapse, n (%)	7 (7)	3 (5)	4 (10)	0.657	0.198
On Cardioactive Medications, n (%)	38 (38)	17 (29)	21 (50)	0.058	3.59
Beta Blockers, n (%)	32 (32)	13 (22)	19 (45)	0.028	4.83
Calcium Channel Blockers, n (%)	7 (7)	5 (9)	2 (5)	0.727	0.122
Antiarrhythmic Drugs, n (%)	3 (3)	2 (3)	1 (2)	1.00	0.00
Duration of Analyzable Holter Data (hours)	193 ± 101	207 ± 101	176 ± 101	0.156	1422
Number of Symptomatic Activations	5.7 ± 4.0	9.7 ± 7.8	13 ± 20	0.524	1127
Atrial Ectopy Percent	1.08 ± 4.42	0.04 ± 0.06	2.51 ± 6.64	<0.001	483
Ventricular Ectopy Percent	0.82 ± 2.91	0.04 ± 0.22	1.88 ± 4.32	<0.001	313

Analysis by age and sex

**Figure 1:**
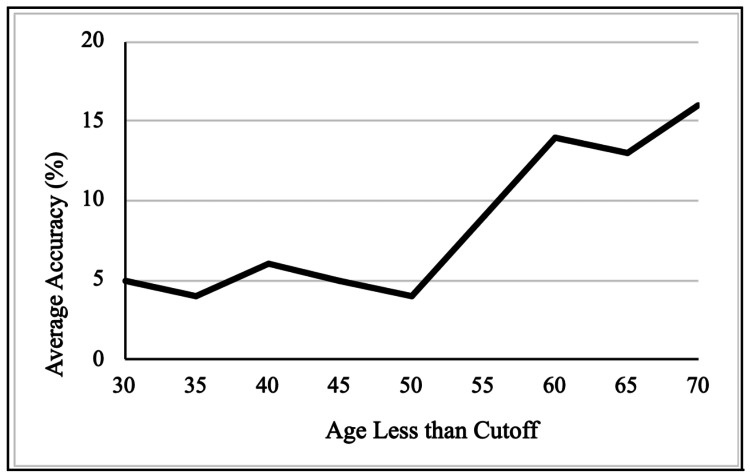
Average accuracy (%) by age less than cutoff

The patients were broken down into age cutoffs, and their respective average accuracies are shown in Table 2 and Figure 1. When stratified by sex (Table 3), there was no statistically significant difference in the number of symptomatic activations, but there was a trend toward higher average accuracy in males, with an average accuracy of 32% compared to their female counterparts at 19% p = 0.080).

**Table 2:** Average accuracy by age cutoffs The test statistic indicates the Wilcoxon Rank Sum test (W statistic) or the Chi-square test statistic when comparing between continuous or categorical variables, respectively.

Age (years)	n (for < age)	Accuracy for < Age (%)	Accuracy for >= Age (%)	p-value	Test statistic
70	81	16	50	<0.001	422
65	69	13	43	< 0.001	596
60	56	14	33	0.003	854
55	43	9	33	< 0.001	761
50	23	4	31	< 0.001	614
45	29	5	29	< 0.001	622
40	24	6	28	0.003	578
35	18	4	26	0.009	476
30	12	5	25	0.05	362

**Table 3:** Summary statistics by sex The test statistic indicates the Wilcoxon Rank Sum test (W statistic) or the Chi-square test statistic when comparing between continuous or categorical variables, respectively.

Variable	Male (N=27)	Female (N=73)	p-value	Test statistic
Age (years)	59 ± 18	54 ± 18	0.258	1132
# Caucasian, (% Caucasian)	22 (82)	44 (60)	0.080	3.06
Number of activations with perceived symptoms	5.0 ± 3.0	5.9 ± 4.4	0.510	901
Number of symptomatic activations with arrhythmias	1.6 ± 2.4	1.2 ± 2.4	0.119	1166
Average Accuracy (%)	32 ± 39	19 ± 30	0.080	1189

Analysis by symptom-arrhythmia association

Patients were also stratified into those who demonstrated symptom-arrhythmia association and those that did not, with the presence of symptom-arrhythmia association defined as accuracy greater than zero. Of the 100 patients, 42 had a symptom-arrhythmia association, and 58 did not. Baseline characteristics, broken down by these two groups, are shown in Table 1. There was no difference in race or comorbidities between the two groups. Patients with symptom-arrhythmia association were more likely to be older (62 vs. 50 years), have a lower left ventricular ejection fraction (56 vs. 63%), and be on cardioactive medications (50 vs. 29%), all P < 0.05 (Table 1).

Among cardioactive medications, those with symptom-arrhythmia association were more likely to be on beta blockers (45 vs 22%), but there was no difference in the other medication types. They also had a higher percentage of atrial (2.51 vs. 0.04%) and ventricular (1.88 vs. 0.04%) arrhythmia as interpreted by the automated analysis over the monitoring period. There were no differences in the percentage of females, percentage of Caucasians, presence of mitral valve prolapse, or the duration of analyzable Holter data in patients with vs. without symptom-arrhythmia correlation. Importantly, there was no difference in the number of symptomatic activations.

The relationship between important predictor variables (age, left ventricular ejection fraction (LVEF), and being on cardioactive medications) and the presence of symptom-arrhythmia association was further explored using logistic regression to adjust for other variables (Table 4). When adjusted for sex, race, and duration of Holter analyzable hours, the variables of age, LVEF, and being on cardioactive medications were still statistically correlated with the presence of symptom-arrhythmia association. This remained true when further adjusted for the presence of mitral valve prolapse or coronary artery disease. When adjusted further for the other important predictor variables, age and LVEF remained correlated with symptom-arrhythmia association, while being on cardioactive medications did not. Neither age nor LVEF remained correlated with symptom-arrhythmia association after adjusting for atrial and ventricular ectopy percent.

**Table 4:** Logistic regression results for the relationship between relevant predictors and the presence of symptom-arrhythmia association Metrics were recorded as odds ratios (95% CI) for logistic regression M1: unadjusted M2: adjusted for sex, race, and duration of Holter analyzable hours M3: M2 + adjusted for presence of mitral valve prolapse, coronary artery disease M4: M3 + adjusted for the other two predictor variables above (age, LVEF, use of cardioactive medications)* M5: M4 + adjusted for atrial ectopy percent, ventricular ectopy percent *for example, when age is used as a predictor variable, M4 includes M3 + adjustment for LVEF and cardioactive medications only

Metric	Predictor Variables	Regression Models				
-	-	M1	M2	M3	M4	M5
Presence of Symptom-Arrhythmia Association	Age	1.05 (1.02 to 1.08)	1.06 (1.03 to 1.09)	1.06 (1.03 to 1.10)	1.06 (1.02 to 1.11)	1.01 (0.96 to 1.06)
	Left Ventricular Ejection Fraction (LVEF)	0.92 (0.85 to 0.97)	0.93 (0.86 to 0.98)	0.93 (0.86 to 0.99)	0.92 (0.85 to 0.98)	0.98 (0.87 to 1.08)
	On Cardioactive Medications	2.86 (1.22 to 6.93)	3.51 (1.42 to 9.13)	3.11 (1.13 to 8.92)	2.18 (0.69 to 6.99)	0.84 (0.07 to 7.31)

**Figure 2:**
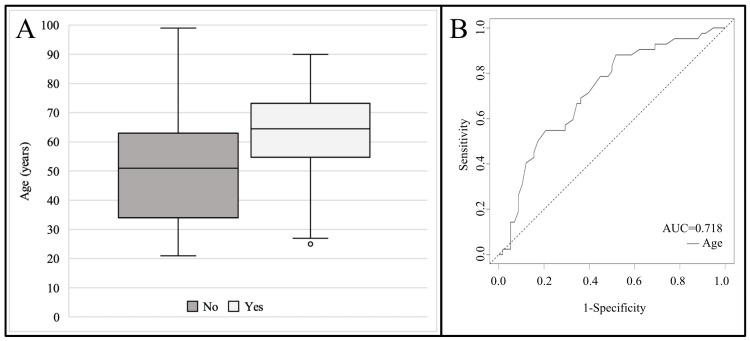
[A] Box plot depicting the age of patients that do not (left) and do (right) demonstrate symptom-arrhythmia association, [B] ROC curve with age as a classifier of the presence of symptom-arrhythmia association

When using age as a classifier of the presence of symptom-arrhythmia association (Figure 2), the ROC curve has an AUC of 0.718 (p < 0.001). The optimal cut-off was age 50 with a sensitivity of 0.881 and a specificity of 0.483.

## Discussion

On average, the probability of a symptomatic event during Holter recording being associated with a true rhythm disturbance was low, at about 22%. When considering the entire Holter monitoring period, only 42% of patients demonstrated any symptom-arrhythmia association. Advancing age was correlated with improved symptom-arrhythmia association, with patients older than 50 years being significantly more likely to show symptom-arrhythmia association as compared to younger patients. Patients older than 70 years had an average accuracy of 50%. Along with increased age, patients who demonstrated symptom-arrhythmia association were more likely to have decreased LVEF. Age and lower ejection fraction being associated with higher diagnostic yield have been demonstrated in prior studies [6]. Importantly, there was no difference in symptom-arrhythmia association by sex, the number of activations, or the presence of MVP.

Potentially, several reasons may have contributed to the overall low accuracy. Holter monitors are often prescribed for palpitations, syncope, and dizziness, among other symptoms. These symptoms can be difficult to differentiate from benign stressors. Additionally, there may have been variability in the Holter device activation instructions given to the patient, as well as the patient’s ability to follow those directions.

While patients with higher symptom-arrhythmia association were more likely to be older, this may have been due to the difference in the underlying burden of arrhythmia. When older patients activated their monitor, there was a higher likelihood of true arrhythmia because they experienced more ectopy at baseline, as one study has demonstrated [8]. In keeping with this, there was a lack of correlation between symptom-arrhythmia association and age after adjusting for atrial and ventricular ectopy burden. This can also be applied to the univariate correlation between symptom-arrhythmia association and decreased LVEF, which was no longer significant after adjusting for atrial and ventricular ectopy burden. While there was a statistically different LVEF in patients with versus without symptom-arrhythmia association, there was no clinical difference since both LVEF values are within normal limits.

This study can help inform Holter prescribing and reading practices. While patients as a whole, and especially young patients, have very low overall accuracy, patients should still be instructed to mark their symptoms. While the chance for an underlying arrhythmia during symptoms is low, reassurance can be a powerful therapy. Further, confirming that patient symptomatology is unrelated to arrhythmia can help avoid unnecessary treatments fraught with their own risks [9]. The results of our study counter the idea that women and patients with a higher number of activations are less accurate. Finally, there was no relationship between symptom-arrhythmia correlation and MVP, although MVP had a low prevalence in the patient population.

There are several limitations to this study. This retrospective study had a relatively small sample size, which limits the generalizability of these findings. The potential unquantifiable variability in instructions given to patients is also a limitation. Patients could have varying thresholds of what they would consider to be positive symptoms, so discrepancies in instructions could result in either over-reporting or under-reporting of symptoms, and hence, the number of activations. There may be “reporting fatigue,” in that patients may report symptoms earlier in the course of the recording, but stop reporting the same symptoms as the days wear on. There may have been errors in the automated reporting of ectopic burden. We were only able to review the provided ECG strips, not the entire recording. Moreover, Holter activations were not broken down by specific symptoms in this study, which limits the ability to identify whether certain cardiac symptoms were more or less likely detectable using Holter recordings. Finally, the results of the analysis by sex may not have achieved statistical significance because of the relatively lower numbers of males in our study population. Future directions for this project include increasing the sample size, providing consistent and uniform instructions to all patients regarding Holter activations, and assessing the efficacy of using Holters to detect specific cardiac symptoms.

## Conclusions

We conclude that symptom-arrhythmia correlation is, in general, poor in human beings, but there are no significant differences in men vs. women. Younger patients may be even less accurate in discerning rhythm disturbances compared to older patients. The results of this study, if validated in additional populations, may provide a cautionary note when prescribing Holter recordings when used for assessment of symptoms that may or may not have an arrhythmic basis.
